# Pros and Cons of In Vitro Methods for Circular RNA Preparation

**DOI:** 10.3390/ijms232113247

**Published:** 2022-10-31

**Authors:** Kyung Hyun Lee, Seongcheol Kim, Seong-Wook Lee

**Affiliations:** 1 R&D Center, Rznomics Inc., Seongnam 13486, Korea; 2 Department of Bioconvergence Engineering, Research Institute of Advanced Omics, Dankook University, Yongin 16890, Korea

**Keywords:** circular RNA, circularization, RNA, RNA vaccine, RNA therapeutics

## Abstract

mRNA is gaining success as a new therapeutic agent and vaccine. However, mRNA has limitations in stability. To overcome the shortcomings of mRNA, circular RNA is emerging as a new modality. In this review, several current methods of manufacturing circular RNA in vitro are introduced and their advantages and disadvantages are reviewed. Furthermore, this study discusses which fields and directions of research and development are needed for the increase in the efficacy and productivity of circular RNA as a therapeutic agent and vaccine formulation.

## 1. Introduction

mRNA is gaining success as a new therapeutic agent and vaccine, such as is the case for COVID-19 vaccines, which were given to hundreds of millions of people [1,2,3]. However, the intrinsically unstable nature of linear mRNA makes it susceptible to ribonuclease activity, which has sparked interest in circular RNA (circRNA), with a covalently joined head (5′) and tail (3′), enabling extended translation for protein synthesis due to its ribonuclease-resistance.

**Figure 1:**
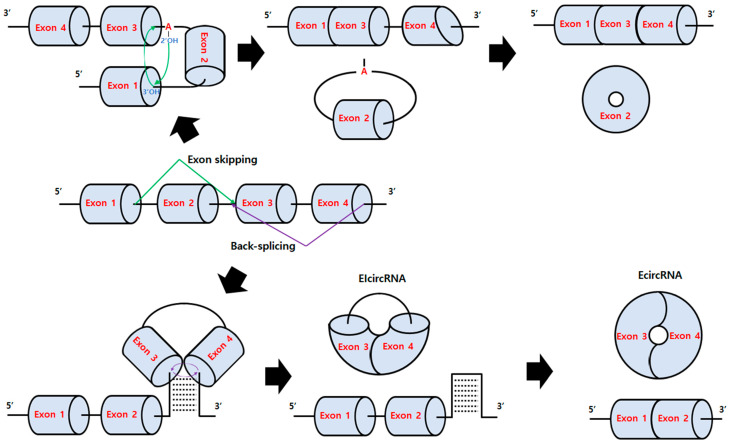
Exon skipping and back-splicing mechanisms [7,12]. For exon skipping (green line), a lariat is created by skipping an exon during the normal splicing mechanism (attack of a 2′-OH group of defined adenosine within the intron onto the 5′-splice site at the 5′ exon (Exon 1) to generate a free 3′-OH, then the nucleophilic attack of the generated 3′-OH onto the 3′-splice). Then, lariat splicing occurs, resulting in Ecircular RNA formation. For back-splicing (purple line), intronic complementary sequences such as Alu elements (abundant transposable elements in primate genome) pair and form an RNA duplex (RNA-binding protein-driven pairing is also possible) that closely juxtapose the splice sites for nucleophilic attack of the 5′-branch point onto a 3′-splice donor and the subsequent attack of the 3′-splice donor onto a 5′-splice acceptor, resulting in the formation of exon-intron circRNA (EIcircRNA) with intron retention or exonic circRNA (EcircRNA) after internal splicing.

CircRNAs are generally produced by exon skipping or back-splicing in organisms in nature and have been reviewed well in several papers [4,5] (Figure 1). Previously, circRNA formation was attributed to mis-splicing [6]. However, it is now believed that circRNAs are generated by specific mechanisms and have various roles in vivo such as sponges of miRNA or protein, cirRNP complexes modulating signaling pathways, and templates for translation [4,5,7], although their roles and related mechanisms are still not fully understood. Some examples of the emerging functions of circRNAs involve aging [8] and cancers [9].

As circRNAs have various application possibilities in biomedical areas, with some advantages compared to linear RNA such as ribonuclease resistance and efficient ribosome recycling due to start and stop codons in close proximity, circRNA has recently gained attention. Therefore, several start-up companies have received investment funds for circRNA technology, and their studies have been more actively published or filed as patents in recent years.

Although in vivo circRNA synthesis is possible for achieving goals for various applications by delivering DNA for transcription and subsequent circRNA formation through back-splicing or other mechanisms [5], in vitro circRNA preparation methods would be more suitable for biomedical applications [4] requiring large-scale production [10,11].

In this review, we will briefly introduce in vitro circRNA preparation techniques for biomedical purposes with recently published examples, then focus on the pros and cons of each method and intensively discuss which fields and direction of research and development should be involved.

## 2. Chemical-Based Methods

**Figure 2:**
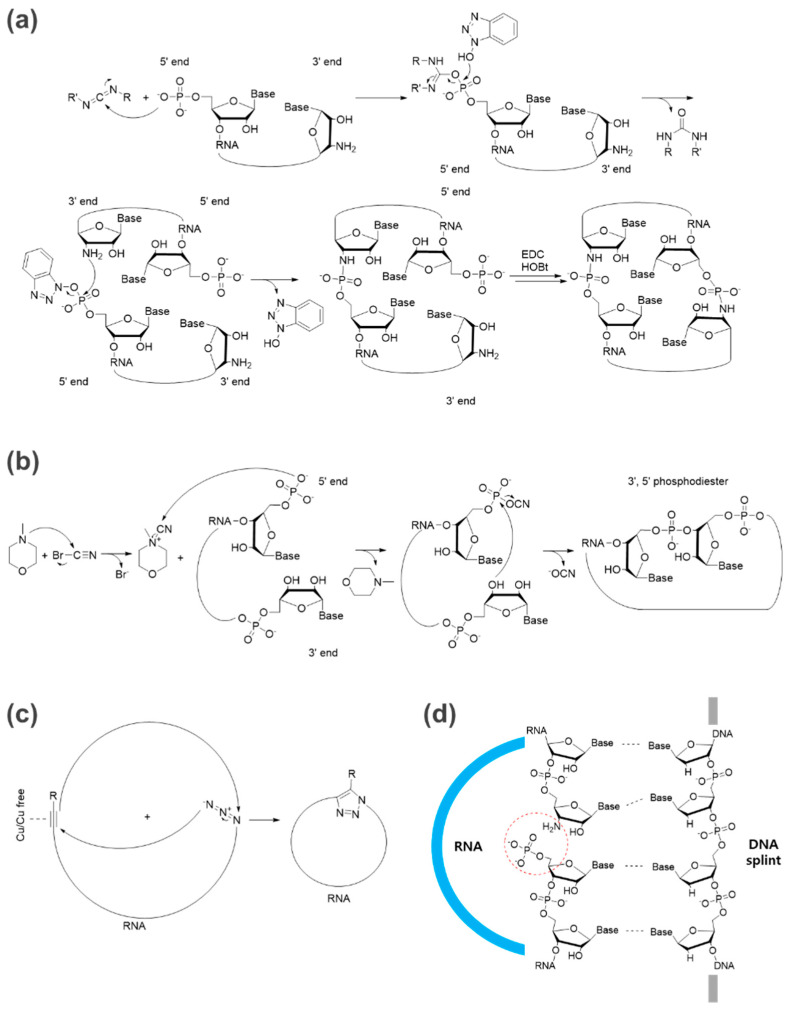
Representative chemical-based methods for circRNA formation. (**a**) An example of an EDC-based reaction [19]. The ligation for circRNA formation between 3′-amino-modified RNA and 5′-phosphorylated RNA can be completed by 1-pot EDC/HOBt chemical synthesis with a DNA splint. (**b**) An example of a BrCN-based reaction [20,21]. (**c**) An example of click chemistry using azide and alkyne for RNA circularization [22]. (**d**) DNA splint-based ligation [23] for pre-orientation of the 5′- and 3′-ends to be linked (red dotted circle, RNA in blue, DNA splint in gray).

Chemical ligation strategies for oligonucleotides have been developed for decades, and examples for circRNA preparation, such as 5′ or 3′ terminal functionalization with amines, thiols, azides, and others for cyclization [11], and representative reactions such as ethyl-3-(3′-dimethylaminopropyl)-carbodiimide (EDC)- or cyanogen bromide (BrCN)-based reactions have been summarized well in previous reviews (Figure 2a,b). Furthermore, simple click chemistry such as azide-alkyne cycloadditions (ACC) is considered a powerful technique for the fast and efficient cyclization of nucleic acids [13] (Figure 2c).

For efficient chemical-based ligation, the ligation junction (5′ and 3′ ends) should be positioned in close proximity by a self-templating effect based on the intrinsic secondary structure [10] or by templating with a DNA splint [14] (Figure 2d) to avoid the formation of intermolecular bonds leading to oligomerization as a side reaction in practical conditions for large-scale circRNA preparation. An intramolecular reaction would be favored over an intermolecular reaction at a lower concentration of RNA substrate, but it is not a practical condition for large-scale production [10]. The DNA splint can be digested by DNase after ligation.

Solid-phase synthesis methods also can be used for circRNA preparation [15]. However, the critical issue is that only relatively short RNA lengths can be synthesized by current solid-phase RNA synthesis methods.

Currently, chemical ligation is not a common method for circRNA preparation due to biosafety concerns and low ligating efficiency compared with other methods such as the permuted intron-exon (PIE) method with nearly 100% efficiency [16,17], as discussed in a previous review [11]. Hopefully, highly efficient biocompatible click chemistry, which is advancing to become more biocompatible, would be a feasible method to overcome biosafety concerns and low ligating efficiency issues [18]. In addition, depending on the chemical reaction, they cannot generate natural 3′,5′-phosphodiester bonds.

## 3. Enzyme-Based Methods

**Figure 3:**
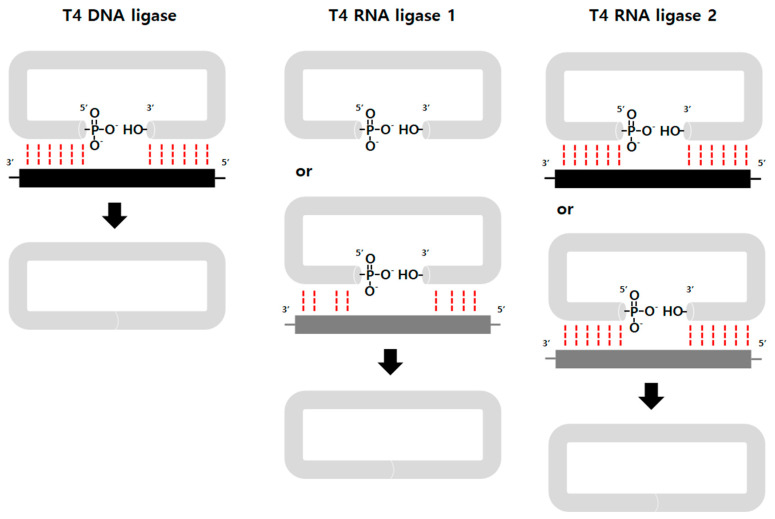
Enzyme-based methods. T4 DNA ligase requires ATP as a cofactor and catalyzes the formation of a phosphodiester bond between a 5′-phosphate and a 3′-hydroxyl group, only in cases of perfect complementarity (red dotted line) between the two strands (RNA in gray, DNA splint in black) at the ligation junction [32]. T4 RNA ligase 1 accepts DNA and RNA fragments as substrates and works on single-stranded substrates with lower reaction specificity [33]. RNA splint (dark gray) or intrinsic secondary structure leaving 2~3 nucleotides unpaired increases efficiency [26,34]. T4 RNA ligase 2 ligates the RNA acceptor strand (3′-OH) with RNA or the DNA donor strand (5′-phosphate) [35]. An RNA or DNA splint can be used to bring the 5′ and 3′ ends together.

Enzyme-based ligation methods have been commonly used for circRNA preparation. Generally, ATP-dependent T4 DNA ligase [24,25], T4 RNA ligase 1 [26], and T4 RNA ligase 2 [27] are used for the reaction and catalyze the joining of RNA termini with 5′ phosphate and 3′ OH groups through three nucleotidyl transfer steps [28]. A DNA splint is also commonly used for efficient enzyme-based ligation, to place 5′ and 3′ ends in close proximity (Figure 3). Potentially, other RNA ligases from various species can also be utilized in the circularization [21].

The ligase has different preferences for the 5′ terminal nucleotide of the donor (pC > pU > pA > pG) and the 3′ terminal nucleotide of acceptor (A > G ≥ C > U) strands [10], which should be taken into account when determining a ligation junction.

Interestingly, recently Liu et al. reported that circRNA prepared by T4 RNA ligase without extraneous fragments exhibited minimized immunogenicity, whereas circRNAs prepared by a ribozyme-based method were immunogenic [29]. The authors claimed that circRNAs prepared by different in vitro methods could induce distinct innate immune responses, even after the purification of circRNAs by high-performance liquid chromatography (HPLC) [30] and polyacrylamide gel electrophoresis (PAGE) methods due to extraneous fragments that form three-dimensional structures. However, as ribozyme-based methods such as the permuted intron-exon (PIE) method [31] generate more RNA contaminants than ligase-based methods using pure linear RNA precursors, it is still not clear whether the RNA contaminants (by-products including fragments derived from ribozyme reaction, etc.) of PIE reactions were 100% eliminated, even after performing HPLC using a size exclusion chromatography column, in which it is not easy to achieve clear separation of RNA peaks, and PAGE purifications. Even femtogram amounts of RNA contaminants can exhibit immunogenicity.

## 4. Ribozyme-Based Methods

### 4.1. PIE (Group I Intron and Group II Intron Self-Splicing Systems)

**Figure 4:**
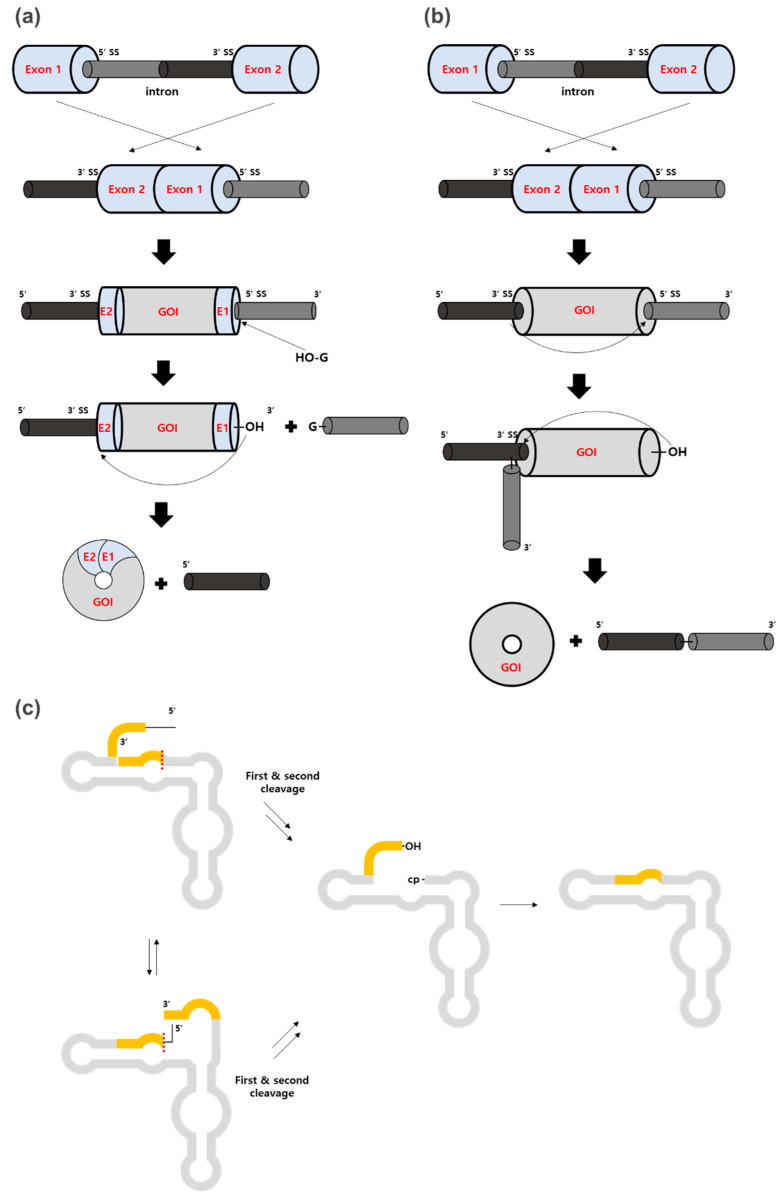
Ribozyme-based methods. (**a**,**b**) The 5′ half of the intron (dark gray) is positioned at the tail of the exon and the remaining 3′ half of the intron (black) is positioned at the head of the exon for both groups I and II intron-based PIE methods. (**a**) For group I intron-based PIE, RNAs transcribed from the permuted intron-exon construct with the gene of interest (GOI) and E1 and E2 fragments undergo circularization by two transesterifications. First, the hydroxyl group of exogenous guanosine (G) attacks at 5′-SS to release the 3′-terminal sequences (5′ half intron). Then, 3′-OH attacks 3′-SS, resulting in the ligation of E1 and E2 to circularize the GOI. (**b**) For group II intron-based PIE, 2′-OH of an internal adenosine attacks 5′-SS to release the 3′-terminal sequences. Then, the 3′-OH terminal residue attacks 3′-SS, resulting in circRNA formation. SS stands for splice site. (**c**) In the hairpin ribozyme-based method, IVT RNA folds into two alternative cleavage-active conformations, cleaving the 3′-end region first and then the 5′-end region second (yellow parts) or vice versa (the red dotted line is the first cleavage in each conformation). The fragments resulting from the first cleavage generate either 2′,3′-cyclic phosphate (cp), or 5′-OH, which are required for the ligation. The final cleavage product can undergo intramolecular ligation to generate circRNA.

PIE methods [31,36] have also been commonly used for circRNA preparation, together with enzyme-based methods. Group I intron or group II intron ribozymes have slightly different mechanisms, but both can be used in the same PIE strategy (Figure 4a,b). Briefly, the 5′ half of the intron is positioned at the tail of the exon and the remaining 3′ half of the intron is positioned at the head of the exon as described in Figure 4a,b. Therefore, exonic circRNA can be generated instead of intronic circRNA which is generated by normal self-splicing. Importantly, the group I intron for PIE introduces the splicing scar (E1 and E2 fragments), which exhibits immunogenicity [29], whereas the group II intron does not. However, the group II intron forms 2′,5′-phosphodiester bonds at the ligation site instead of natural 3′,5′-phosphodiester bonds, which is controversial as the precise mechanism is unclear [10,21,37].

Compared to chemical- or enzyme-based ligations, large-size RNAs can be circularized using ribozyme-based methods. For example, Wesselhoeft et al. reported that circRNA up to 5 Kb in length could be prepared by PIE in vitro [17]. Moreover, circRNA prepared by the PIE method with the internal ribosome entry site (IRES) and optimized accessory sequences showed robust and stable protein expression in eukaryotic cells [17]. More importantly, circRNA has reduced immunogenicity and can extend translation duration in vivo [38]. Interestingly, unmodified exogenous circRNA is able to bypass cellular RNA sensors that provoke immune responses in cells and mice, while previous reports suggested that N^1^-methylpseudouridine (m1ψ) for linear RNAs [39] or N6-methyladenosine (m6A) for linear and circRNAs [40] were important for preventing exogenous RNAs from activating Toll-like receptors (TLRs) and RIG-1 [41], as well as for translation [42,43]. Therefore, using unmodified circRNAs would be a promising strategy for further applications, and could avoid patent-related issues on RNA modifications, although that is still not conclusive, and m6A is generally used for circRNA preparation [44].

Recently, circRNA prepared by PIE was applied for vaccine development for SARS-Cov2 and variants [45,46]. In these studies, circRNA vaccines elicited neutralizing antibodies and T cell responses by expressing antigens such as the trimeric receptor binding domain (RBD) of the spike protein or a new version of a spike trimer. In addition to higher stability and antigen-encoding efficiency, circRNA vaccines elicited higher proportions of neutralizing antibodies and distinct Th1-skewed immune responses [45] compared to a linear m1ψ -modified mRNA vaccine.

As another example, circRNAs prepared by PIE- or enzyme-based methods were used to prepare adenosine deaminase acting on RNA (ADAR which is for the precise conversion of adenosine to inosine)-recruiting RNAs (arRNAs) for higher editing efficiency and longer periods of RNA editing than their linear counterparts [47], demonstrating the applicability of circRNAs to various biomedical areas and purposes.

However, as RNA structures for ribozymes or IRES functions are crucial in PIE-based methods, spacer sequences between functional regions or other accessory sequences should be finely tuned for the proper folding of ribozymes for PIE reactions and IRESs for subsequent efficient translation [44].

### 4.2. Hairpin Ribozyme

Self-cleaving ribozymes were also used for circularization in vitro [27,48] through the intrinsic cleavage and ligation activities of hairpin ribozymes (HPRs) and hammerhead ribozymes from the genomes of viroids and the hepatitis delta virus [49]. In this case, in vitro transcription (IVT) using circular single-stranded DNA templates generated multimeric repeating RNAs harboring the HPR elements, then RNA can cleave itself into monomer length. Subsequently, circularization occurred by intrinsic HPR activity [50] (Figure 4c). However, only a few examples of circRNA preparation for short RNAs have been reported so far [27,51], and circRNA is not stable due to the dynamic equilibrium of HPR-catalyzed cleavage and ligation [10].

## 5. Discussion

Here, we briefly reviewed the representative in vitro methods for circRNA preparation. Each method has pros and cons as the ligation strategies are completely different (Table 1) and there is much room for improvement in each.

To improve circRNA preparation strategies, we would like to discuss several issues and further efforts that should be undertaken by researchers.

Firstly, in vitro transcribed RNAs have several issues that affect in vitro circularization efficiency, such as heterogeneity of the 3′ ends of IVT RNAs and aggregates upon precipitation [53]. These issues were reviewed well with some examples to address them in a previous paper [54]. For example, the heterogeneity issue of the 3′ ends of IVT RNAs can be resolved using ribozyme technology, which can trim the ends and generate homogeneous IVT RNA ends [55].

Circularization efficiency is also strongly dependent on the RNA structure [56]. Therefore, a DNA splint strategy can be used to prevent the RNA substrate from folding into an unsuitable structure for chemical- or enzyme-based methods [56]. Ribozyme-based methods are strongly structure-dependent as the structure of ribozyme should be properly folded for proper activity and should target the specific ligation junction site on the RNA structure, which is unique for each GOI and affects ligation efficiency.

For ribozyme-based methods, antisense and antisense binding sequences (homologous arms at the 5′ and 3′ ends) [17] can work like the DNA splint strategy in chemical or enzyme-based methods. Although accurately predicting RNA structure is still challenging, information on the intrinsic secondary structure of RNA for positioning the 5′ and 3′ ends in close proximity would be still helpful for more efficient ribozyme-based ligation.

Chen and Lu suggested that unnatural bases or RNA-binding proteins (RBPs) may help to improve the efficiency of ligation by altering the structure of linear RNA precursors [11]. However, in PIE methods, modified bases may negatively affect the ligation efficiency due to changed RNA folding, affecting ribozyme activity, as well as stability [44], and immunogenicity, which is still controversial for circRNA [38].

For in vivo applications, circRNA purity is an important factor as some reaction contaminants such as dsRNA or DNA can trigger unwanted immunogenicity. For example, TLRs in endosomes and RIG-I-like receptors (RLRs) in cytoplasm detect nonself RNA in endosomes, initiating an inflammatory cascade as well as cytoplasmic dsRNA-responding proteins such as protein kinase R (PKR) [57]. Generally, native or denatured PAGE or HPLC purification is used to purify RNAs. However, PAGE purification is not optimal for large-scale production and purification processes. As RNase R is not sufficient to obtain pure circRNAs due to some highly structured resistant species, HPLC purification without RNase R treatment would be best. For the PIE reaction, the current HPLC protocol for circRNA purification using a size-exclusion column would still be insufficient as the method cannot clearly separate many different RNA species from the PIE reaction mixture. Therefore, the purification methodology should be further improved.

In the case of preparing circRNA with PIE methods, the intronic scar should be also considered in terms of immunogenicity [29] and translation efficiency [44], while other methods do not add extra sequences to circRNA.

For biomedical applications, a large-scale production process is required. This scale-up issue was previously discussed by Chen and Lu [11]. As suggested, an increase in linear precursors for the circularization reaction will lead to an increase in side reactions, resulting in a lower yield of circRNA and difficulty in purification. To address the issue, the authors suggested using new types of reactions such as microreactors.

In addition to method optimization, the translation efficiency or function of the prepared circRNA should be comparable with or better than that of its linear modified RNA counterpart by further optimization as shown in recent papers [44,45,47].

Finally, to develop new strategies for circRNA preparation, we would like to suggest that several requirements mentioned below should be met, in addition to previous suggestions [10,11].

(1) Less circularization and purification steps for circRNA preparation are required. Even an ethanol precipitation step affects RNA’s quality, suggesting that any additional steps would affect the efficiency and yield of circRNA preparation. The ideal method would be efficient circularization during IVT without additional steps. In this case, a ribozyme-based method would be more suitable.

(2) In the case of developing ribozyme-based methods, the intronic scar should be eliminated or minimized to prevent immunogenicity from the intronic scar. As the intronic scar can affect translation efficiency in addition to immunogenicity, the surrounding sequences should be optimized to avoid those effects [44]. If a novel ribozyme-based strategy could be developed in which circRNA does not contain intronic scars, the optimization process will be much easier for each circRNA with different GOIs and elements, and additional sequences, such as spacers, will not be required to avoid the effects of intronic scars.

(3) During circRNA preparation and purification processes, nicked circRNA populations [17] should be decreased as much as possible.

(4) The efficacy of larger GOI circularization should be improved. As the circularization of linear precursors, especially for large sizes, is entropically disfavored, a ribozyme-based method using an intrinsic self-splicing mechanism would be advantageous over chemical- and enzyme-based methods for preparing large circRNAs [10].

(5) Large-scale production and purification should be possible. If circularization can be completed during IVT as a one-step process, the IVT scale would be proportional to circRNA production.

Although major in vitro circRNA preparation methods were developed in the 1990s [23,24,31,58,59], technological advances for in vitro preparation methods for circRNA have not progressed much. Even though those methods have successfully been used for various applications, new strategies with better efficiency and fewer drawbacks, such as simple and large-scale production processes for circRNA, should be developed to meet commercial use requirements.

## Figures and Tables

**Table 1:** Pros and cons of each circRNA preparation method for practical use.

Method	Pros	Cons
Chemical-based ligation	• Various chemical reactions	• Toxicity concerns
		• Uses DNA splint
	• No intronic scar	• Multiple steps • Unnatural bond
Enzyme-based ligation	• No intronic scar (however, the first 2~3 nucleotides are guanosine by T7, SP6, T3 RNA polymerase)	• Uses DNA or RNA splint • Ligases from T4 bacteriophage [52] require 5′-monophosphate
	• Large GOI	• Multiple steps • Ligation efficiency
Ribozyme-based ligation	• Relatively simple • Large GOI	• Intronic scar by group I intron PIE • Larger transcript including ribozyme
	• Reactions in vitro & in vivo	• RNA contaminants after reaction

## Data Availability

Not applicable.
